# Supplementary material for: The Novel Immune Checkpoint GPR56 Is Expressed on Tumor-Infiltrating Lymphocytes and Selectively Upregulated upon TCR Signaling
Source: Cancers (Basel). 2022 Jun 28;14(13):3164. doi: 10.3390/cancers14133164 (PMC9264967; doi:10.3390/cancers14133164)
Supplement: Supplementary file 1 [file cancers-14-03164-s001.zip › cancers-1732753-supplementary.pdf]

cancers

MDPI

Supplementary Materials: The Novel Immune Checkpoint GPR56 Is Expressed on Tumor-Infiltrating Lymphocytes and Selectively Upregulated upon TCR Signaling

Vrouyr Bilemjian, Martijn R. Vlaming, Jimena Álvarez Freile, Gerwin Huls, Marco De Bruyn and Edwin Bremer

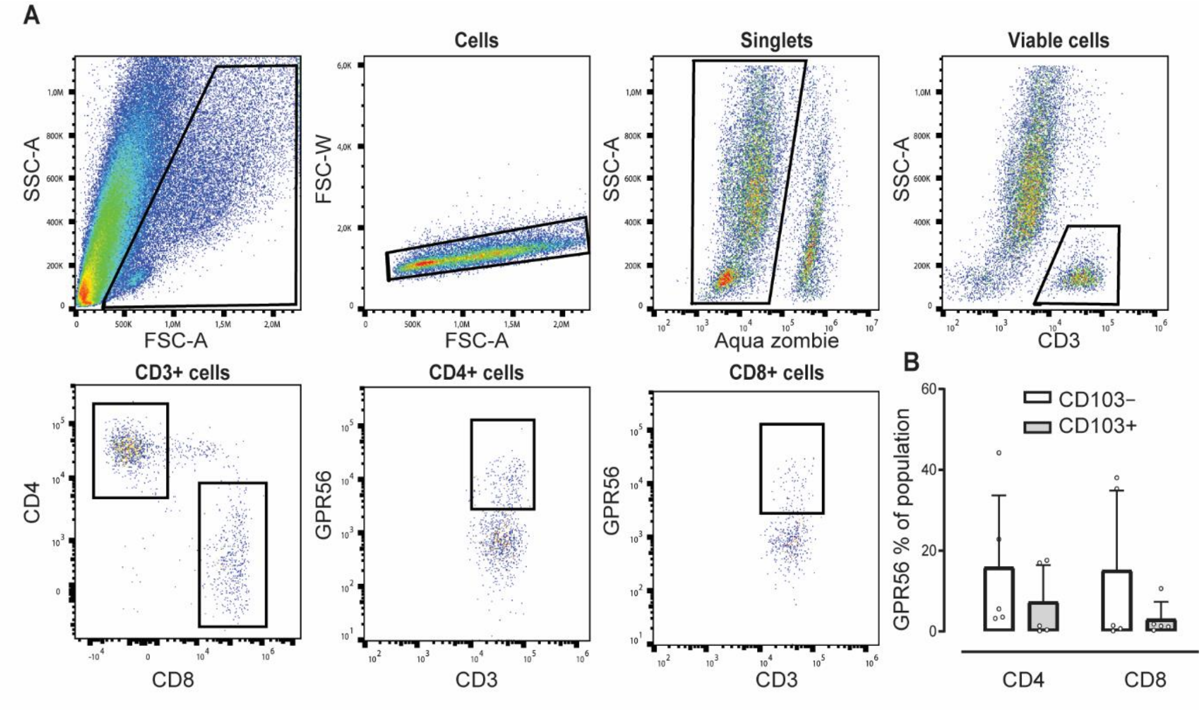

Figure S1. Gating strategy used to define GPR56+ CD4+ cells and GPR56+ CD8 + TILs. (**A**) Gating strategy used to define GPR56+ CD4+ cells and GPR56+ CD8+ TILs. (**B**) % of cells positive for GPR56 within the CD103− and the CD103+ TIL population (*n* = 5).

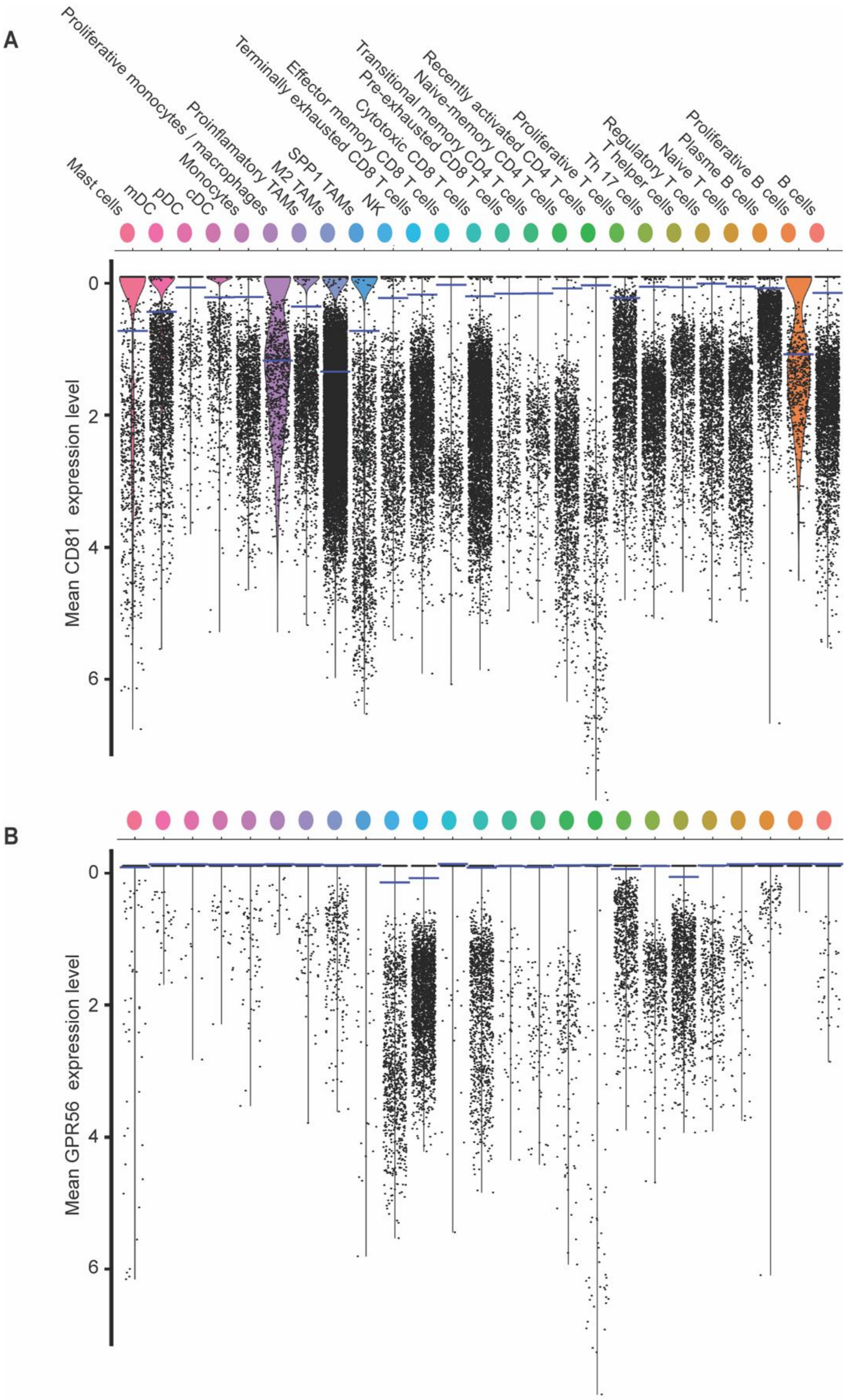

Figure S2. CD81 is expressed on many immune cell populations within the tumor. (**A**) Displaying the different CD81 expressing tumor infiltrating immune-cell subtypes. (**B**) Displaying the different GPR56 expressing tumor infiltrating immune-cell subtypes with mean expression values indicated with a blue line.

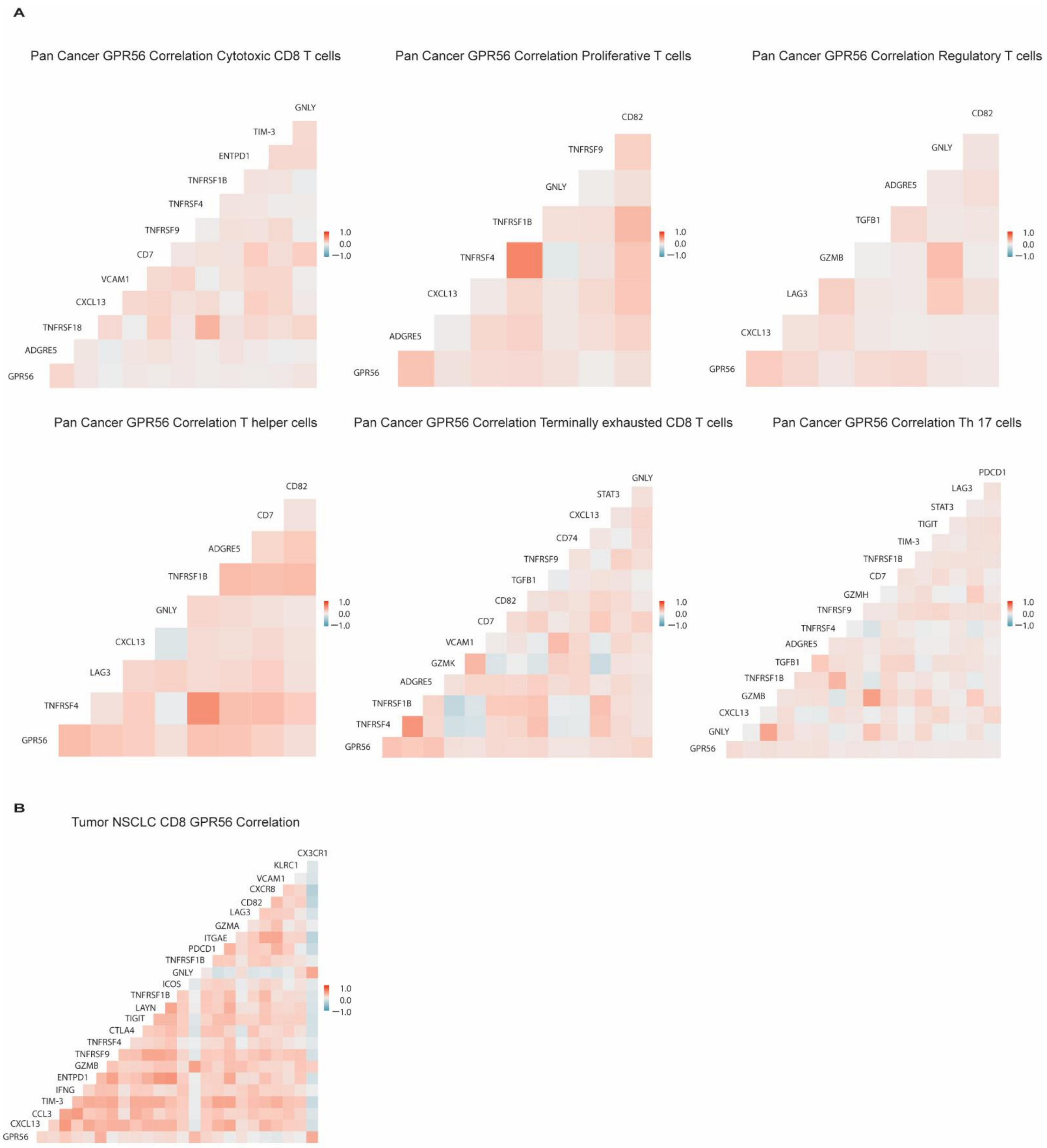

Figure S3. GPR56 is correlated with subset of genes within the different tumor infiltrating immune-cell subtypes. (**A**) Displaying GPR56 correlation with different genes within the tumor infiltrating immune-cell subtypes from Single-cell tumor immune atlas RNA sequencing data-set. We used ggcorr function to do pair wise pearson correlation coefficient calculation for different genes and plot the data in the form of correlation matrix. (**B**) Displaying GPR56 correlation with different genes within the tumor infiltrating CD8 lymphocytes from NSCLC single-cell RNA sequencing data set.
